# New Mitogenomes from the Genus *Ablabesmyia* (Diptera: Chironomidae, Tanypodiinae): Characterization and Phylogenetic Implications

**DOI:** 10.3390/insects16020178

**Published:** 2025-02-07

**Authors:** Wen-Bin Liu, Wen-Xuan Pei, Ya-Ning Tang, Jia-Xin Nie, Wei Cao, Cheng-Yan Wang, Chun-Cai Yan

**Affiliations:** Tianjin Key Laboratory of Conservation, Utilization of Animal Diversity, Tianjin Normal University, Tianjin 300387, China; skylwb@tjnu.edu.cn (W.-B.L.);

**Keywords:** chironomids, mitogenome, phylogenomics, *Ablabesmyia*, Tanypodiinae

## Abstract

(1) Background: The insect mitogenome encodes essential genetic components and serves as an effective marker for molecular identification and phylogenetic analysis in insects due to its small size, maternal inheritance, and rapid evolution. The morphological identification of *Ablabesmyia* is challenging, particularly for non-experts. Thus, there is an increasing need for molecular data to improve classification accuracy and phylogenetic analysis. (2) Methods: Our analysis encompassed eight species of *Ablabesmyia*, a single species of *Conchapelopia*, one species of *Denopelopia*, and one species of *Thienemannimyia*, all originating from China. We then performed a comprehensive analysis of the nucleotide composition, sequence length, and evolutionary rate. (3) Results: All newly assembled mitogenomes displayed a negative GC-skew, indicating a cytosine bias, while most exhibited a positive AT-skew, reflecting an adenine and thymine abundance. All thirteen protein-coding genes (PCGs) featured the conventional start codon ATN, aligning closely with the typical mitochondrial start codon observed in insects. The evolutionary rates of these PCGs can be ordered as follows: *ND2* > *ATP8* > *ND6* > *ND4* > *ND5* > *ND3* > *ND4L* > *ND1* > *CYTB* > *COIII* > *ATP6* > *COII* > *COI*. (4) Conclusions: These newly sequenced mitogenomes exhibit structural features and nucleotide compositions that closely align with those of previously reported Chironomidae species, marking a significant expansion of the chironomid mitogenome database.

## Simple Summary

Chironomidae, a highly diverse family of freshwater flies, possess an unparalleled ability to thrive in a vast array of aquatic environments. The Chironomidae family is currently divided into 11 subfamilies, with the subfamily Tanypodinae comprising nearly 60 genera and over 600 species worldwide, and the genus *Ablabesmyia* being one of the largest within the Pentaneurini tribe. The mitochondrial genome of the genus *Ablabesmyia* has not yet been reported. We have undertaken the sequencing, assembly, and annotation of the mitochondrial genomes for a selection of species within the genus *Ablabesmyia*, which includes eight distinct species. Our comprehensive examination, which includes a total of 17 mitochondrial genomes, offers unprecedented insights into the evolutionary narratives of these taxa, contributing significantly to the field of entomology and evolutionary biology.

## 1. Introduction

Recently, insect mitochondrial genomes (mitogenomes) have attracted increased research attention for their remarkable conservation in structure and gene arrangement, mirroring those of their ancestral forms [1,2,3,4,5]. The insect mitogenome, a double-stranded circular molecule of 14–20 kb, encodes essential genetic components and serves as an effective marker for molecular identification and phylogenetic analysis in Insecta due to its small size, maternal inheritance, and rapid evolution [6,7,8,9,10].

Chironomidae, a highly diverse family of freshwater midges, possess an unparalleled ability to thrive in a vast array of aquatic environments, ranging from oxygen-depleted waters to the frigid peaks of the Himalayas and the deep abyss of Lake Baikal [11]. Their remarkable resilience in extreme conditions, such as temperatures as low as −16 °C, and their status as one of the most widely distributed insects globally, make them invaluable bioindicators for assessing ecological health and detecting environmental changes [12,13]. With an estimated 15,000 species worldwide, these aquatic insects exhibit exceptional species diversity, which can be attributed to their ancient lineage, limited dispersal, and evolutionary flexibility [11]. They play a crucial role in aquatic ecosystems by significantly contributing to the processing of detritus and influencing trophic dynamics. Their tolerance to harsh conditions also renders them valuable for ecological and water quality assessments [14]. Furthermore, their high population densities and unique life cycle characteristics are central to theoretical ecological studies and have practical applications in biological monitoring and as a food source for various animals [15,16].

The Chironomidae family is currently classified into 11 subfamilies within the global taxonomy system [17,18]. Among these subfamilies, Orthocladiinae, Tanypodinae, and Chironominae are particularly notable, as they encompass the greatest number of species and have a broad distribution across the globe [19]. The subfamily Tanypodinae, comprising nearly 60 genera and over 600 species worldwide, is divided into eight tribes, with the largest being the tribe Pentaneurini [20,21]. The genus *Ablabesmyia*, one of the largest within the Pentaneurini tribe, was established by Johannsen in 1905, with *Tipula monilis* Linnaeus 1758 designated as the type species [22].

The genus *Ablabesmyia*, featuring four subgenera—*Ablabesmyia* Johannsen, *Asayia* Roback, *Karelia* Roback, and *Sartaia* Roback, is distinguished among Chironomidae by the unique characteristics of its adult genitalia [23,24]. The male *Ablabesmyia* are easily recognized by their vibrant pigmentation, which adorns their leg bands and wings, and further distinguished by the unique arrangement of acrostichal setae that diverge around the prescutellar depression, framing the medial scar—a hallmark of the genus [25]. During the pupal stage, the genus *Ablabesmyia* stands out with a distinctive set of features, including a robust thoracic horn, a pronounced thoracic comb, and an adhesive sheath encasing the anal macrosetae [26]. The larvae of *Ablabesmyia* are marked by several distinctive traits: typically, one to three dark posterior parapod claws, a segmentation of the basal segment of the maxillary palp into two to six parts with the ring organ positioned between the two most distal segments, and pecten hypopharyngeal teeth that are notably unequal in size, all of which serve as diagnostic characteristics for this genus [27]. The larvae of *Ablabesmyia* are eurytopic, living in a variety of small and large lotic and lentic waters, bog pools, the littoral part of eutrophic lakes, and in rivers [22,27]. The morphological identification of *Ablabesmyia* can be quite challenging, especially for those without expert knowledge. Therefore, there is a growing need for additional molecular data to facilitate accurate classification and to enhance phylogenetic analysis.

In order to elucidate the mitochondrial genomes of the genus *Ablabesmyia* and to deepen our understanding of the intricate phylogenetic relationships within the family Chironomidae, we have undertaken the sequencing, assembly, and annotation of the mitochondrial genomes for a selection of species within the genus *Ablabesmyia*, which includes eight distinct species. Additionally, we have included the mitogenomes of one species each from *Conchapelopia*, *Denopelopia*, and *Thienemannimyia*. To enrich our analysis and provide a more profound comprehension of the mitogenome’s characteristics, we have also incorporated six mitogenomes that have been published previously. Leveraging the strength of Bayesian inference (BI) and maximum likelihood (ML) methods, we have conducted a thorough analysis across various databases. This approach has allowed us to unravel the complex phylogenetic relationships within the subfamily Tanypodinae. Our comprehensive examination, which includes a total of 17 mitochondrial genomes, offers unprecedented insights into the evolutionary narratives of these taxa, contributing significantly to the fields of entomology and evolutionary biology.

## 2. Materials and Methods

### 2.1. Taxon Sampling and Sequencing

Our analysis encompassed eight species of *Ablabesmyia*, a single species of *Conchapelopia*, one species of *Denopelopia*, and one species of *Thienemannimyia*, all originating from China, with detailed information provided in Table 1. Additionally, for the purposes of comparative mitogenomic analysis and phylogenetic reconstruction, we retrieved the mitogenomes of *Clinotanypus yani* Cheng & Wang, 2008 and *Tanypus punctipennis* Meigen, 1818 from GenBank [16,28]. Building upon extensive prior phylogenetic research conducted on Chironomidae, we selected four species that served as outgroups from each of the closely related genera: *Cricotopus dentatus* Hirvenoja, 1985, *Cricotopus bicinctus* (Meigen, 1818), *Boreoheptagyia alulasetosa* Makarchenko, Wu & Wang, 2008, and *Boreoheptagyia kurobebrevis* (Sasa & Okazawa, 1992). Before DNA extraction and morphological examination, all samples were preserved in a solution containing 85% to 95% ethanol and stored at a temperature of −20 °C.

To extract total genomic DNA, we utilized the TIANamp Genomic DNA Kit (DP304; TIANGEN, Beijing, China). The voucher specimens have been deposited at the College of Life Sciences, Tianjin Normal University, Tianjin, China, for potential future reference and investigation. The whole genome samples were then sent to Berry Genomics in Beijing, China, for sequencing. For library preparation, we employed the TruSeq Nano DNA HT Sample Preparation Kit from Illumina (United States Illumina Company, San Diego, CA, USA). Sequencing was performed on the Illumina NovaSeq 6000 platform (United States Illumina Company, San Diego, CA, USA) using a paired-end strategy (PE150), targeting DNA fragments with an insert size of 350 bp. After sequencing, raw reads were processed using Trimmomatic, and the resulting clean reads were retained for further downstream analyses [29].

### 2.2. Assembly, Annotation, and Composition Analyses

To reconstruct the mitogenome sequences, we utilized NOVOPlasty v3.8.3 (Brussels, Belgium) in a de novo assembly approach, starting with the COI gene as the seed sequence and exploring a spectrum of k-mer sizes ranging from 23 to 39 bp to optimize the mitogenome assembly [30]. Annotation of the mitogenome adhered to the protocol outlined by [31], with enhancements. Specifically, the secondary structure of tRNAs was deciphered using the MITOS WebServer, while rRNAs and protein-coding genes (PCGs) were annotated manually within Geneious, leveraging the Clustal Omega algorithm for alignment [32]. To further validate and refine the boundaries of rRNAs and PCGs, we employed the Clustal W function available in MEGA 11. The analysis of nucleotide composition bias and the composition of individual genes was conducted using SeqKit v0.16.0, a tool developed in Chongqing, China [33]. Visualization of the mitochondrial genome map was accomplished using the CGView server, which is available at https://cgview.ca/ (accessed on 15 July 2024) [34]. MEGA 11 was utilized to determine the nucleotide composition, codon usage, and relative synonymous codon usage of the mitochondrial genome [35]. Quantification of nucleotide composition bias was achieved through the calculation of AT-skew, defined as (A − T)/(A + T), and GC-skew, calculated as (G − C)/(G + C). Furthermore, we computed the synonymous (Ks) and non-synonymous substitution rates (Ka) using DnaSP6.0 [34], providing insights into the evolutionary dynamics of the mitogenome.

### 2.3. Phylogenetic Analyses

To construct phylogenetic trees, we selectively isolated two ribosomal RNA (rRNA) genes and thirteen protein-coding genes (PCGs) from seventeen mitochondrial genomes. Sequence alignment for both nucleotides and proteins was carried out using MAFFT (Osaka, Japan), employing the L-INS-I algorithm to exclude ambiguous alignments. Trimming of sequences was then executed with Trimal v1.4.1 (Barcelona, Spain), preparing the sequences for phylogenetic analysis. This analysis was based on five distinct data matrices generated by FASconCAT-G v1.04 (Santa Cruz, CA, USA), specifically configured as follows: (1) cds_fna, encompassing all codon positions of the 13 PCGs; (2) cds_rna, integrating all codon positions of the 13 PCGs along with the two rRNA sequences; (3) cds12_rna, incorporating the first and second codon positions of the 13 PCGs and the two rRNA sequences; (4) cds12, focusing solely on the first and second codon positions of the 13 PCGs; and (5) cds_faa, utilizing the amino acid sequences derived from the 13 PCGs. To quantify the variability among these matrices, we utilized AliGROOVE v1.06 (Bonn, Germany), drawing inspiration from previous studies conducted by [35,36,37,38]. For the ML analysis, the optimal substitution models are used for each gene partition. The bootstrapping phase and node support were calculated by using 1000 replicates. Following this, maximum likelihood (ML) trees were constructed using IQ-tree v2.0.7, and Bayesian inference (BI) trees were constructed using Phylobayes-MPI v1.8, providing robust insights into the evolutionary relationships among the mitochondrial genomes under investigation.

## 3. Results and Discussion

### 3.1. Mitogenomic Organization

**Figure 1:**
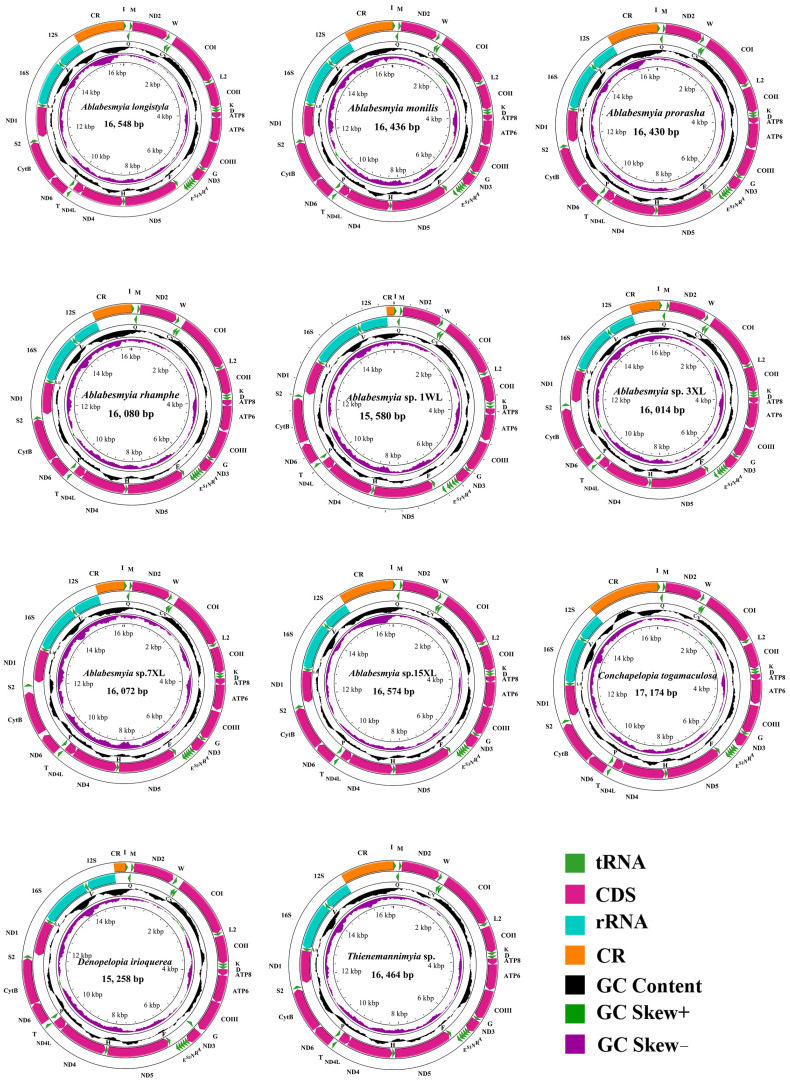
The mitochondrial genome (mitogenome) map of various representative species spanning four genera within the subfamily Tanypodiinae, highlighting their distinctive attributes. Standard abbreviations are used for protein-coding genes (PCGs) and ribosomal RNAs (rRNAs), while single-letter abbreviations are employed for transfer RNAs (tRNAs) to ensure clarity. The second concentric circle highlights the GC content of the entire mitogenome, providing insights into its nucleotide composition. The third circle illustrates the GC-skew, offering additional information about the nucleotide distribution. Finally, the innermost circle represents the total length of the mitogenome, providing a comprehensive overview of its characteristics.

The sequences newly acquired exhibited a range of lengths, varying from 15,258 base pairs (bp) in *Denopelopia irioquerea* to 17,174 bp in *Conchapelopia togamaculosa*. The primary factor contributing to this variation was the fluctuating size of the control region (CR), which ranged from 191 bp in *Ablabesmyia* sp. 1WL to 1959 bp in *Ablabesmyia* sp. 3XL (Table 2). All of the newly assembled mitogenomes contained a standard complement of genetic elements, consisting of one control region (CR) and 37 genes. Specifically, these genes encompassed 13 protein-coding genes (PCGs), 22 transfer RNAs (tRNAs), and 2 ribosomal RNAs (rRNAs), as depicted in Figure 1. Notably, the lengths of the majority of these newly assembled mitogenomes were comparable to those observed in previously published Chironomidae mitogenomes. The sequence characteristics of the represented species are visually summarized in Figure 1.

**Figure 2:**
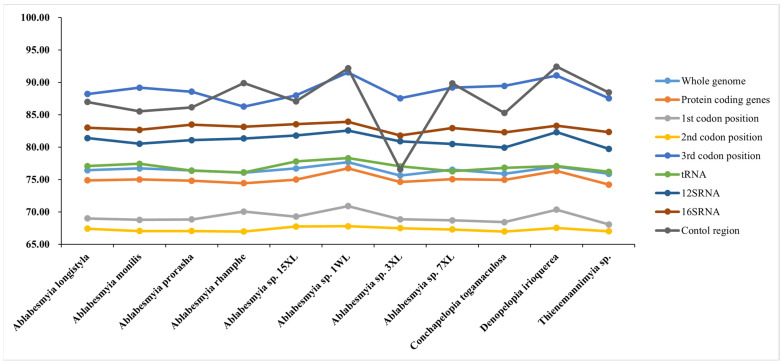
Difference in AT content of protein-coding genes of Tanypodiinae mitogenomes.

The nucleotide composition of the newly reported mitogenomes demonstrated consistent patterns among the samples, as presented in Table 2, which were characteristic of the AT-bias typical in Chironomidae and other insect lineages. Significant variation was observed in the AT content of the mitochondrial genomes, with values ranging from 74.20% in *B. alulasetosa* to 78.78% in *C. bicinctus* (Figure 2 and Table 2). Notably, the control region (CR) exhibited the highest AT content, ranging from 76.57% in *A.* sp. 3XL to 92.41% in *D. irioquerea*. In contrast, the AT content in transfer RNAs (tRNAs) and protein-coding genes (PCGs) was relatively lower compared to ribosomal RNAs (rRNAs) (Table 2).

All newly assembled mitogenomes displayed a negative GC-skew, indicating a cytosine bias, while most exhibited a positive AT-skew, reflecting an adenine and thymine abundance. The GC-skew ranged from −0.195 in *A*. sp. 15XL to −0.060 in *C. yani*. The AT-skew varied from 0.006 in *C. bicinctus* to 0.051 in *A*. sp. 3XL, with two exceptions: *C. yani* and *B. kurobebrevis*, which displayed negative AT-skews of −0.130 and −0.008, respectively. The GC content itself ranged from 21.22% in *C. bicinctus* to 25.80% in *B. alulasetosa*, offering additional insights into the nucleotide composition of these mitogenomes (Table 2).

### 3.2. Protein-Coding Genes, Codon Usage, and Evolutionary Rates

Among diverse species, no substantial differences were observed in the size of transfer RNA (tRNA), protein-coding genes (PCGs), and ribosomal RNA (rRNA). In particular, the total length of the thirteen PCGs in the acquired mitogenomes varied minimally, ranging between 11,210 and 11,225 base pairs. A comparative analysis of our results with existing Chironomidae data revealed a significant pattern: the adenine–thymine (AT) content at the third codon positions of the PCGs was notably higher compared to those at the first and second positions (Figure 2). Notably, a majority of the seventeen mitogenomes exhibited a negative guanine–cytosine (GC)-skew in their PCGs. Additionally, all these mitogenomes displayed a negative AT-skew in the same genes, with values ranging from −0.195 in *B. kurobebrevis* to −0.155 in *A*. sp. 1WL. The AT content percentage varied from 70.90% in *B. alulasetosa* to 76.73% in *A*. sp. 1WL, whereas the GC content ranged from 23.27% in *A.* sp. 1WL to 29.10% in *B. alulasetosa* (see Table 2 for detailed information).

**Figure 3:**
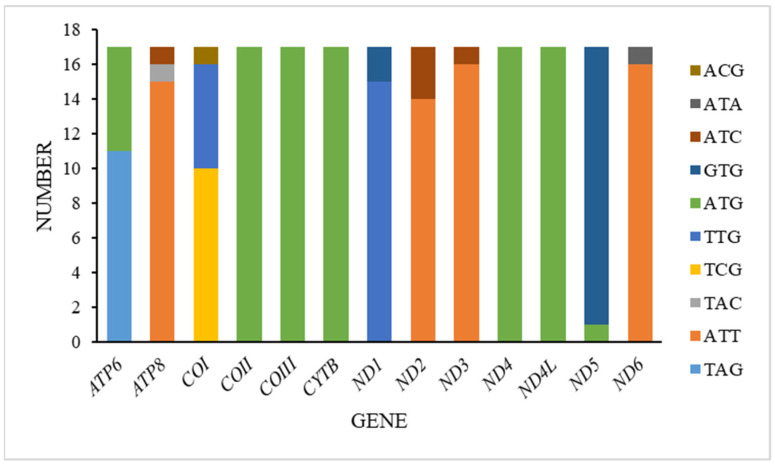
Start codons of protein-coding genes among Tanypodiinae mitogenomes.

In the mitogenomes we acquired, all thirteen protein-coding genes (PCGs) mostly featured the conventional start codon ATN, aligning closely with the typical mitochondrial start codon observed in insects. However, deviations were noted in other genes. Specifically, the cytochrome oxidase subunit I (*COI*) gene utilized TCG as its start codon in ten species, TTG in six species, and ACG in one species. The ATP synthase subunit 8 (*ATP8*) gene began with ATT in fifteen species and ATC in one species, while the ATP synthase subunit 6 (*ATP6*) gene started with ATG in all species. The NADH dehydrogenase subunit 1 (*ND*1) gene utilized TTG as its start codon in fifteen species and GTG in two species. Similarly, the NADH dehydrogenase subunit 2 (*ND2*) gene started with ATT in fourteen species and ATC in three species, and the NADH dehydrogenase subunit 3 (*ND3*) gene began with ATT in sixteen species and ATC in one species. Furthermore, the cytochrome oxidase subunit 2 (*COII*), cytochrome oxidase subunit 3 (*COIII*), cytochrome b (CYTB), NADH dehydrogenase subunit 4 (ND4), and NADH dehydrogenase subunit 4L (*ND4L*) genes consistently started with ATG. The NADH dehydrogenase subunit 5 (*ND5*) gene uniquely started with GTG in sixteen species and ATG in one species, while the NADH dehydrogenase subunit 6 (*ND6*) gene exclusively began with ATT in sixteen species and ATA in one species (Figure 3).

Regarding stop codons, the majority of the 13 PCGs primarily utilized TAA. Exceptions included the *COI* gene with twelve instances of TAA and one TAG, the *COIII* gene with thirteen TAA, the *ND6* gene with one TAA and one TAG, the *ND1* gene with one TAG, and the *ND4* gene with five TAG instances.

**Figure 4:**
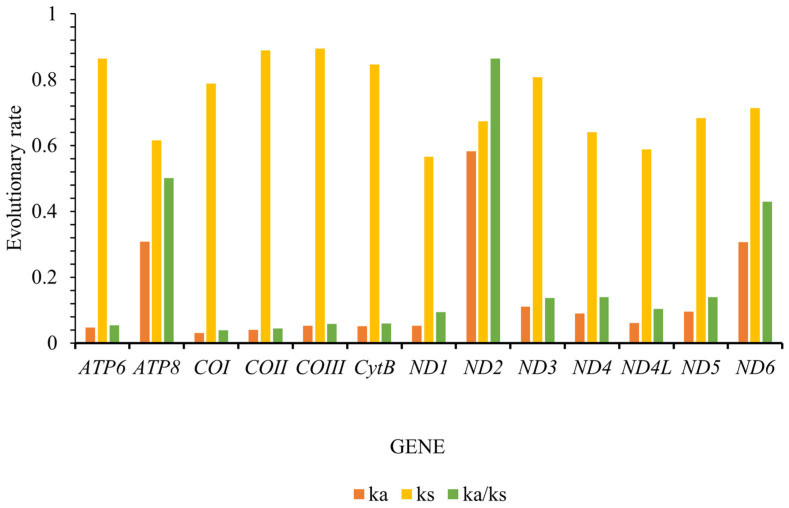
Evolutionary rates of the 13 protein-coding genes (PCGs) in Tanypodiinae mitogenomes. Non-synonymous substitutions are denoted as Ka, while synonymous substitutions are represented as Ks. The Ka/Ks ratio indicates the selection pressure on each PCG. The *x*-axis lists the 13 PCGs, and the *y*-axis displays the Ka/Ks values.

The Ka/Ks ratio, also known as ω, is a widely utilized metric to quantify the rate of sequence evolution in the context of natural selection. Our findings closely mirror those reported for other insect species, demonstrating that the Ka/Ks values for all thirteen protein-coding genes (PCGs) consistently fell below one, with a range spanning from 0.039 for cytochrome oxidase subunit 1 (*COI*) to 0.864 for NADH dehydrogenase subunit 2 (*ND2*) (Figure 4). The evolutionary rates of these PCGs can be ordered as follows: *ND2* > *ATP8* > *ND6* > *ND4* > *ND5* > *ND3* > *ND4L* > *ND1* > *CYTB* > *COIII* > *ATP6* > *COII* > *COI*. Our findings notably demonstrate that many of these genes have evolved under purifying selection, which acts to remove harmful mutations and is influenced by varying selective pressures. Specifically, the low ω values observed for *COII* and *COI* indicate a stringent selection environment, suggesting strong evolutionary constraints. In contrast, the higher ω values for *ND2*, *ATP8*, and *ND6* suggest a more relaxed purifying selection pressure, implying that these genes may be evolving with greater freedom (Figure 4). These insights enhance our understanding of the evolutionary dynamics of these PCGs and the role of natural selection in shaping their sequence evolution.

The lengths of the seventeen mitochondrial tRNAs exhibited considerable variation, ranging from 1480 to 1525 base pairs (bp). The AT content of these tRNAs also showed significant differences, with values spanning from 75.40% in *B. alulasetosa* to 80.00% in *C. bicinctus*. Notably, all tRNAs, except for *B. kurobebrevis* (with an AT-skew of −0.108) and *C. bicinctus* (with an AT-skew of −0.007), demonstrated a positive AT-skew, with values ranging from 0.008 to 0.049. In contrast to the AT content, the GC content of the tRNAs varied from 20.00% in *C. bicinctus* to 24.60% in *B. alulasetosa*. Furthermore, the GC-skew displayed substantial variability, with values ranging from 0.129 in *A. prorasha* to 0.185 in *C. dentatus*. These findings highlight the diverse nucleotide compositions and skewness patterns within the mitochondrial tRNAs of the studied species.

Regarding the rRNA sequences, their lengths exhibited considerable variation, ranging from 2161 bp in *B. alulasetosa* to 2255 bp in *A*. sp. 1WL. The AT content remained consistently high across all mitogenomes, with values varying between 79.75% and 83.54%. In contrast, the GC content showed a narrower range, spanning from 16.46% to 20.35%. Notably, the GC-skew of all mitogenomes, except for *C. yani,* which exhibited a negative value of −0.310, was significantly positive, ranging from 0.025 to 0.295. While the majority of mitogenomes demonstrated a negative AT-skew, ranging from −0.028 to −0.001, four species—*C. yani*, *C. bicinctus*, *B. alulasetosa*, and *B. kurobebrevis*—displayed positive AT-skews, with values of 0.015, 0.002, 0.013, and 0.011, respectively. For a detailed summary of these findings, please refer to Table 2.

### 3.3. Phylogenetic Relationships

**Figure 5:**
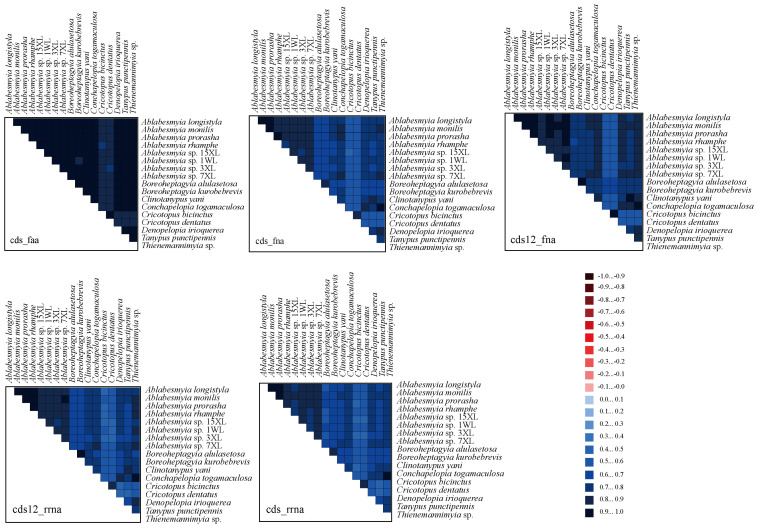
The evaluation of heterogeneity among the mitogenomes of 17 species within the subfamily Tanypodiinae focused on their protein-coding genes (PCGs), amino acid sequences, and ribosomal RNAs (rRNAs). Sequence similarity was visually depicted using colored blocks, based on AliGROOVE scores that range from −1 (indicating significant heterogeneity between datasets, shown in red) to +1 (indicating minimal heterogeneity, shown in blue). A lighter color in each dataset’s block corresponds to greater heterogeneity, while a darker color indicates lower heterogeneity.

The analysis of heterogeneity divergence differences provides insights into the similarities of mitochondrial gene sequences across various species. Notably, due to the redundancy of the genetic code, the AA dataset exhibited the lowest level of heterogeneity, while the cds12_rrna dataset showed a relatively higher degree of heterogeneity (Figure 5). This indicates that the mutation rate at the third codon position in protein-coding genes (PCGs) exceeds that of the first and second positions. As a result, the third codon positions are deemed unsuitable for inferring phylogenetic relationships within the genus *Ablabesmyia*. Additionally, the heterogeneity observed in the outgroup species of *Cricotopus* and *Boreoheptagyia* is significantly lower than that in the ingroup species.

**Figure 6:**
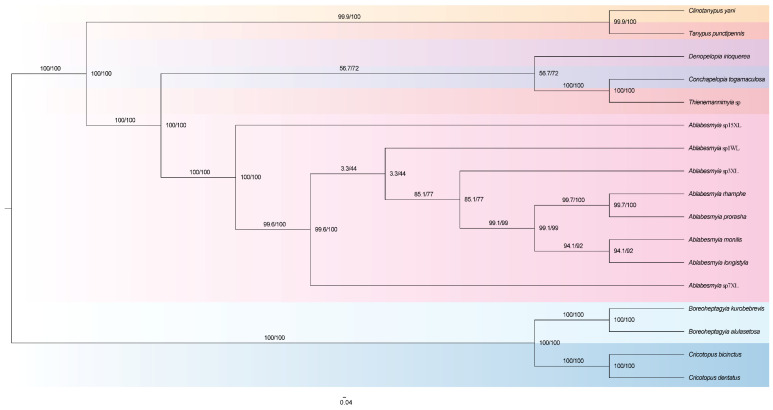
Phylogenetic ML tree of the genus *Ablabesmyia*, based on analysis PCG12_rRNA in partition.

Both Bayesian inference (BI) and maximum likelihood (ML) analyses of these ten datasets consistently identified a similar topological structure across the phylogenetic trees, although there were differences in branch lengths and statistical support values (Figures S1–S9). From the perspective of mitochondrial genomes, the eight species of genus *Ablabesmyia* identified morphologically are supported as belonging to this genus, and existing data also support the division of this genus into several subgenera (Figure 6).

The study of the phylogeny of the family Chironomidae has made some progress based on female adults and other morphological characteristics [40,41]. In recent years, the development of molecular biology has enabled further exploration of the phylogenetic relationships within the subfamily Tanypodinae [19]. In the phylogenetic analysis of Chironomidae using molecular data from fragments of 18S rRNA, 28S rRNA, CAD1, CAD4, and mtCOI, analyzed with mixed-model Bayesian and maximum likelihood methods, the results show that *Ablabesmyia* and *Conchapelopia* are more closely related and form a sister group with *Tanypus*, a finding further supported by our research on mitochondrial genomes [19]. Cladistic analyses using 86 morphological characters from 115 species have confirmed that the species *Ablabesmyia*, *Denopelopia*, *Conchapelopia*, and *Thienemannimyia* are closely related, forming a group referred to as the tribe Pentaneurini, and the tribes Tanypodini + Clinotanypodini are sister groups to the tribe Pentaneurini, which is well supported by mitochondrial genome studies [42]. Incorporating sequence data from mitochondrial COI and nuclear 28S and CAD, Bayesian and maximum likelihood phylogenetic inferences support the view that *Ablabesmyia* is a terminal group, representing branches or evolutionary stages that appeared later in the evolutionary process, within the tribe Pentaneurini, a finding also reflected in our mitochondrial genome data [43]. To more precisely define and evaluate the phylogenetic relationships within the subfamily Tanypondinae, it is essential to incorporate mitogenomes from a wider variety of species across different genera.

## 4. Conclusions

Despite the distinct morphological traits observed among the developmental stages—larvae, pupae, and adult males and females—of different genera of Tanypodinae, there is a notable discrepancy between phylogenetic results based on morphology, short gene sequences, and mitochondrial genome data. However, an emerging consensus from molecular phylogenetics underscores the enduring importance of morphological analysis in the study of chironomids. Moreover, while the comprehensive analysis of mitochondrial genomes offers exciting possibilities, it requires rigorous examination and careful consideration. A holistic systematic analysis that integrates morphological, biogeographical, and life history traits across various developmental stages of insects, supplemented by genomic data, is essential. Such an integrative approach is likely to illuminate the intrinsic evolutionary connections within the natural world.

For the first time, the mitochondrial genomes of eight species within the genus *Ablabesmyia* have been meticulously annotated, assembled, and documented. These newly sequenced mitogenomes exhibit structural features and nucleotide compositions that closely align with those of previously reported Chironomidae species, marking a significant expansion of the chironomid mitogenome database. This advancement lays a solid foundation for future phylogenetic studies. The newly sequenced mitogenomes show remarkably similar structural traits and nucleotide compositions, closely matching the data previously published for Chironomidae.

## Figures and Tables

**Table 1:** Collection information of newly sequenced species in this study.

Species	Sample ID	Location	Longitude and Latitude	Date	Collector
*Ablabesmyia longistyla*	ZLS001	Li shan, Shanxi, China	N35°39′11″, E111°96′10″	25 July 2022	Yao Yuan
*Ablabesmyia monilis*	BBa005	Ningyuan, Tianjin, China	N39°17′02″, E117°21′33″	25 October 2022	Du Xiaohui
*Ablabesmyia prorasha*	2BW006	Bawangling National Forest Park, Hainan, China	N19°08′86″, E109°12′92″	3 April 2008	Yan Chuncai
*Ablabesmyia rhamphe*	2CB223	Five Fingers Group Scenic Area, Hainan, China	N18°75′30″, E109°54′71″	23 July 2017	Yan Chuncai
*Ablabesmyia* sp. 15XL	WRJ001	Rongjiang, Guizhou, China	N25°93′18″, E108°52′18″	21 April 2015	Liu Wenbin
*Ablabesmyia* sp. 1WL	RGN037	Nanling National Forest Park, Guangdong, China	N24°94′03″, E113°04′87″	26 July 2021	Caowei
*Ablabesmyia* sp. 3XL	SGL001	Guilin, Guangxi, China	N25°88′10″, E110°49′43″	28 July 2022	Yan Chuncai
*Ablabesmyia* sp. 7XL	SHC001	Luocheng, Hechi, Guangxi, China	N24°77′79″, E108°86′61″	11 April 2015	Liu Wenbin
*Conchapelopia togamaculosa*	RGN037	Nanling National Forest Park, Guangdong, China	N24°94′03″, E113°04′87″	24 July 2021	Caowei
*Denopelopia irioquerea*	TES001	Seven Sisters Mountains, Enshi, China	N30°02′40″, E109°75′07″	9 July 2015	Song Chao
*Thienemannimyia* sp.	3HB001	Menyuan, Haibei, Qinghai, China	N37°38′62″, E101°61′06″	10 July 2018	Yan Chuncai

**Table 2:** Nucleotide composition of 17 mitogenomes.

**Species**	**Whole Genome**					**PCG**					**tRNA**				
	**Length (bp)**	**AT%**	**AT-Skew**	**GC%**	**GC-Skew**	**Length (bp)**	**AT%**	**AT-Skew**	**GC%**	**GC-Skew**	**Length (bp)**	**AT%**	**AT-Skew**	**GC%**	**GC-Skew**
*Ablabesmyia longistyla*	16,548	76.42	0.034	23.55	−0.172	11,222	74.86	−0.165	25.13	−0.019	1508	77.05	0.017	22.95	0.150
*Ablabesmyia monilis*	16,436	76.71	0.015	23.28	−0.160	11,222	74.99	−0.172	25.00	−0.018	1509	77.41	0.026	22.60	0.161
*Ablabesmyia prorasha*	16,430	76.38	0.030	23.61	−0.166	11,216	74.81	−0.167	25.19	−0.025	1505	76.35	0.034	23.66	0.129
*Ablabesmyia rhamphe*	16,080	76.05	0.040	23.94	−0.170	11,213	74.42	−0.163	25.58	−0.010	1505	76.08	0.017	23.92	0.133
*Ablabesmyia* sp. 15XL	16,574	76.70	0.044	23.25	−0.195	11,224	74.98	−0.165	25.02	−0.010	1516	77.77	0.011	22.23	0.157
*Ablabesmyia* sp. 1WL	15,580	77.68	0.034	22.32	−0.191	11,210	76.73	−0.155	23.27	−0.034	1525	78.29	0.017	21.70	0.154
*Ablabesmyia* sp. 3XL	16,014	75.61	0.051	24.35	−0.173	11,219	74.62	−0.158	25.38	−0.036	1480	77.02	0.044	22.98	0.147
*Ablabesmyia* sp. 7XL	16,072	76.51	0.035	23.48	−0.181	11,225	75.04	−0.163	24.95	−0.029	1509	76.27	0.015	23.72	0.151
*Conchapelopia togamaculosa*	17,174	75.88	0.029	24.09	−0.155	11,219	74.94	−0.171	25.05	−0.016	1504	76.80	0.008	23.20	0.141
*Denopelopia irioquerea*	15,258	76.97	0.009	23.03	−0.160	11,217	76.29	−0.164	23.71	−0.017	1495	77.06	0.002	22.95	0.131
*Thienemannimyia* sp.	16,464	75.87	0.038	24.13	−0.167	11,219	74.20	−0.168	25.80	−0.024	1499	76.18	0.021	23.81	0.154
*Clinotanypus yani*	16,247	75.00	−0.130	25.00	−0.060	11,215	72.50	−0.190	27.50	0.000	1490	75.70	0.020	24.30	−0.120
*Tanypus punctipennis*	16,215	75.70	0.044	24.40	−0.172	11,222	72.70	−0.177	27.30	0.033	1484	76.10	0.007	23.80	0.160
*Cricotopus dentatus*	15,547	77.20	0.017	22.79	−0.117	11,214	74.96	−0.183	25.04	0.015	1481	79.61	0.004	20.40	0.185
*Cricotopus bicinctus*	16,198	78.78	0.006	21.22	−0.128	11,223	76.10	−0.172	23.90	0.036	1490	80.00	−0.007	20.00	0.161
*Boreoheptagyia alulasetosa*	16,411	74.20	0.016	25.80	−0.116	11,210	70.90	−0.185	29.10	−0.007	1482	75.40	0.005	24.60	0.138
*Boreoheptagyia kurobebrevis*	16,409	77.20	−0.008	22.80	−0.100	11,215	74.70	−0.195	25.30	0.020	1486	76.20	−0.108	23.80	0.151
**Species**	**rRNA**					**CR**					**GenBank Accession**			**Reference**	
	**Length (bp)**	**AT%**	**AT-Skew**	**GC%**	**GC-Skew**	**Length (bp)**	**AT%**	**AT-Skew**	**GC%**	**GC-Skew**					
*Ablabesmyia longistyla*	2201	82.03	−0.058	17.97	0.244	1310	86.95	0.052	13.05	−0.333	PQ323369			This Study	
*Ablabesmyia monilis*	2203	81.76	−0.071	18.25	0.265	1421	85.50	0.111	14.29	−0.488	PQ323370			This Study	
*Ablabesmyia prorasha*	2193	82.27	−0.070	17.73	0.253	1383	86.12	0.097	13.89	−0.343	PQ323371			This Study	
*Ablabesmyia rhamphe*	2194	82.23	−0.090	17.78	0.288	1047	89.88	0.014	10.02	−0.295	PQ323372			This Study	
*Ablabesmyia* sp. 15XL	2196	82.66	−0.061	17.34	0.247	1461	87.06	0.261	12.39	−0.790	PQ323378			This Study	
*Ablabesmyia* sp. 1WL	2255	83.23	−0.060	16.77	0.295	191	92.15	0.148	7.85	−0.468	PQ323375			This Study	
*Ablabesmyia* sp. 3XL	2204	81.33	−0.084	18.67	0.285	1959	76.57	0.101	23.28	−0.241	PQ323376			This Study	
*Ablabesmyia* sp. 7XL	2197	81.70	−0.063	18.30	0.272	747	89.83	0.031	10.04	−0.253	PQ323377			This Study	
*Conchapelopia togamaculosa*	2179	81.10	−0.051	18.90	0.281	794	85.26	0.235	13.98	−0.369	PQ323373			This Study	
*Denopelopia irioquerea*	2184	82.79	−0.072	17.21	0.269	277	92.41	0.039	7.58	−0.237	PQ323374			This Study	
*Thienemannimyia* sp.	2179	81.02	−0.058	18.98	0.269	1348	88.42	0.032	11.57	−0.411	PQ323379			This Study	
*Clinotanypus yani*	2176	80.20	0.015	19.80	−0.310	1095	88.70	−0.080	11.30	−0.390	MW373524			[16]	
*Tanypus punctipennis*	2187	79.90	−0.057	20.05	0.294	948	91.60	0.002	8.40	−0.500	MZ475054			[28]	
*Cricotopus dentatus*	2181	82.55	−0.017	17.38	0.241	409	89.98	0.109	10.02	−0.689	OP006251			[39]	
*Cricotopus bicinctus*	2181	83.54	0.002	16.46	0.273	1067	91.10	−0.023	8.90	−0.242	OP006255			[39]	
*Boreoheptagyia alulasetosa*	2161	79.75	0.013	20.35	0.241	1261	87.70	0.040	12.30	−0.220	MZ043574			[16]	
*Boreoheptagyia kurobebrevis*	2170	79.90	0.011	20.10	0.025	1309	91.30	−0.021	8.70	−0.299	MZ043576			[16]	

## Data Availability

The following information was supplied regarding the availability of DNA sequences: The new mitogenomes were deposited in GenBank of the NCBI and the accession numbers are in Table 2.

## Supplementary Materials

The following supporting information can be downloaded at: https://www.mdpi.com/article/10.3390/insects16020178/s1. Figures S1–S4 present ML phylogenetic trees of the genus *Ablabesmyia* based on various analyses (cds_faa, cds_rrna, cds12_fna, cds-fna) using the Partition model in IQTREE, with support values indicated by SHaLRT/UFBoot2, respectively. Figures S5–S9 present BI phylogenomic trees of the genus *Ablabesmyia* based on various analyses (cds_faa, cds_fna, cds_rrna, cds12_fna, cds12_rrna) using the CAT + GTR model in phylobayes, with support values on nodes indicated by Bayesian posterior probabilities. Table S1 presents the final gene partitions for the maximum likelihood phylogenetic analysis.
