# Supplementary material for: New Mitogenomes from the Genus Ablabesmyia (Diptera: Chironomidae, Tanypodiinae): Characterization and Phylogenetic Implications
Source: Insects. 2025 Feb 7;16(2):178. doi: 10.3390/insects16020178 (PMC11856422; doi:10.3390/insects16020178)
Supplement: Supplementary file 1 [file insects-16-00178-s001.zip › Table S1.pdf]

Table S1. Final gene partitions for the Maximum Likelihood Phylogenetic analysis

Matrixs	Partition names	Best model
cds_faa	*ATP6, ND1, ND4, ND4L, ND5*	mtInv+R4
	ATP8, ND2, ND3, ND6	mtMet+F+R4
	COI, COII, COIII, CYTB	mtZOA+R3
cds_fna	*ATP6, COI, COII, COIII, CYTB, ND3*	GTR+F+R4
	ATP8, ND2, ND6	TVM+F+I+G4
	ND1, ND4, ND4L, ND5	TVM+F+R4
cds_rrna	*ATP6, COI, COII, COIII, CYTB, ND3*	GTR+F+R4
	ATP8, ND2, ND6, ND1, ND4, ND4L, ND5	TVM+F+R4
	l-rRNA, s-rRNA	GTR+F+I+G4
cds12_fna	*ATP6, COI, COII, COIII, CYTB*	TIM2+F+I+G4
	ATP8, ND2, ND3, ND6	TVM+F+I+G4
	ND1, ND4, ND4L, ND5	GTR+F+I+G4
cds12_rrna	*ATP6, COI, COII, COIII, CYTB*	TIM2+F+I+G
	ATP8, ND2, ND3, ND6	TVM+F+I+G4
	ND1, ND4, ND4L, ND5, l-rRNA, s-rRNA	GTR+F+I+G4
